# Formulation and Evaluation of Turmeric- and Neem-Based Topical Nanoemulgel against Microbial Infection

**DOI:** 10.3390/gels10090578

**Published:** 2024-09-05

**Authors:** Sumon Giri, Anhic Chakraborty, Chiranjit Mandal, Tushar Kanti Rajwar, Jitu Halder, Zainab Irfan, Mostafa M. Gouda

**Affiliations:** 1 Department of Pharmaceutical Technology, Brainware University, Barasat, Kolkata 700125, India; sumongiri610@gmail.com (S.G.); anhicchakraborty.2001@gmail.com (A.C.); chiranjitsunny100@gmail.com (C.M.); 2 Department of Pharmaceutics, School of Pharmaceutical Sciences, Siksha ‘O’ Anusandhan (Deemed to be University), Bhubaneswar 751030, India; tusharkantirajwar23497@gmail.com (T.K.R.); jituhalder18@gmail.com (J.H.); 3 College of Biosystems Engineering and Food Science, Zhejiang University, Hangzhou 310058, China; 4 Department of Nutrition & Food Science, National Research Centre, Dokki, Giza 12622, Egypt

**Keywords:** nanoemulsion, nanoemulgel, herbal plants, microbial infection, topical application

## Abstract

The combination of nanoemulgel and phytochemistry has resulted in several recent discoveries in the field of topical delivery systems. The present study aimed to prepare nanoemulgel based on turmeric (*Curcuma longa*) and neem (*Azadirachta indica*) against microbial infection as topical drug delivery. Olive oil (oil phase), Tween 80 (surfactant), and PEG600 (co-surfactant) were used for the preparation of nanoemulsion. Carbopol 934 was used as a gelling agent to convert the nanoemulsion to nanoemulgel and promote the control of the release of biological properties of turmeric and neem. The nanoemulsion was characterized based on particle size distribution, PDI values, and compatibility using FTIR analysis. In contrast, the nanoemulgel was evaluated based on pH, viscosity, spreadability, plant extract and excipient compatibility or physical state, in vitro study, ex vivo mucoadhesive study, antimicrobial properties, and stability. The resulting nanoemulsion was homogeneous and stable during the centrifugation process, with the smallest droplets and low PDI values. FTIR analysis also confirmed good compatibility and absence of phase separation between the oil substance, surfactant, and co-surfactant with both plant extracts. The improved nanoemulgel also demonstrated a smooth texture, good consistency, good pH, desired viscosity, ex vivo mucoadhesive strength with the highest spreadability, and 18 h in vitro drug release. Additionally, it exhibited better antimicrobial properties against different microbial strains. Stability studies also revealed that the product had good rheological properties and physicochemical state for a period of over 3 months. The present study affirmed that turmeric- and neem-based nanoemulgel is a promising alternative for microbial infection particularly associated with microorganisms via topical application.

## 1. Introduction

The skin, the largest organ in the body, provides vital support to the defense mechanism of the body [1]. Skin layers have the most significant ability to shield the body from microbial communities, ultraviolet (UV) radiation, and direct sunlight [2]. Since it serves as the body’s initial line of defense against the infiltration of harmful substances like medications and other chemical compounds, researchers must focus on addressing this issue [3]. Skin diseases are becoming increasingly more dangerous since millions of people are reported to be suffering from a number of them [4]. Skin infections can be caused by several pathogenic organisms but mostly by bacteria and fungi [5]. Skin infections and illnesses may expose people to danger if they remain untreated. Fortunately, there are several advantages to using this route of drug delivery over other conventional methods, including reduced systemic drug interactions, reduced invasiveness, simplicity in administration, avoidance of first-pass effects, extended duration of medication release, and improved patient compliance [6].

In recent years, the healthcare research community has focused more on topical and transdermal application strategies because of their potential to treat systemic or skin disorders, ability to carry a specific drug when in contact with skin, high bioavailability, minor side effects, and regulated drug delivery profiles [7,8]. Transdermal drug delivery aims to enhance percutaneous absorption by crossing the stratum corneum [9]. Molecules can be encapsulated in nanocarriers, including nanoemulsions, which may be rendered soluble as a formulation strategy. A nanoemulsion is made up of three components: water, oil, and an emulsifying agent (surfactant). It is a kind of colloidal carrier system as well as a lipid-based delivery nanosystem [10,11]. It has been claimed that the use of nanoemulsion can result in improved drug penetration through the skin. These biphasic nanosystems provide enhanced skin penetration via drug-loaded nanoscale droplets (<100 nm) that readily permeate skin layers because of the medicine’s high concentration gradient which reduces toxicity and causes no irritation [12]. By increasing the surface area accessible for interaction with biological membranes, these nano-sized droplets improve absorption and bioavailability. They tend to require external energy to prepare, and the mixture that is turned out is usually transparent [13]. The co-surfactant and surfactant (o/w or w/o type) work together to stabilize nanoemulsion globules [14,15]. Water and alcohol are examples of aqueous solvents that comprise the nanoemulsion’s aqueous phase, whereas synthetic or natural lipids are often utilized as the oil phase unless the oil itself serves as an active agent [16].

Hydrated skin permits greater payloads, and gel networks provide easy and regulated migration of nanovesicles from the matrix to the skin’s surface. Carbomers, Pluronic’s, xanthan gum, and carrageenan are examples of biocompatible polymers that have been used as gel systems to transdermally administer emulsified medications [17]. Its low viscosity limits the application of the nanoemulsion in treating a range of skin conditions. [18]. Nanosized emulsions combined into a gel-based system make up nanoemulgels. Using a gelling ingredient that is used to prepare the gel basis, nanoemulgel transforms nanoemulsions into a denser, more stable, and non-sticky system [19]. To increase the designed nanoemulsion’s viscosity for transdermal administration, Carbopol 934 is reported to be frequently utilized [18]. The nanoemulsion is changed into a nanoemulgel by adding a gel matrix to it indicating that the transdermal applications benefitted more from the nanoemulgel.

Natural products have a significant impact on treating a variety of ailments with greater safety and fewer side effects, which is the reason they are widely used as alternative medications today [20]. Turmeric and neem are two of the most notable herbal plants utilized for their abundance of bioactive components and centuries-old traditional medical use [21]. Turmeric, belonging to the Zingiberaceae family and well known for its active constituent Curcumin, possesses a wide range of biological properties such as antimicrobial and antioxidant properties [22,23]. Neem, belonging to the family of Meliaceae, is native to the Indian subcontinent and a few African countries. Neem is a multipurpose tree having excellent antibacterial, antifungal, antioxidant, anti-inflammatory, and insecticidal characteristics due to the plethora of active phytoconstituents such as nimbidin, nimbin, salannin, margolone, and azadirachtin [24,25]. Based on their pharmacological properties reported, it can be assumed that a combination of turmeric and neem would show a synergistic effect.

These naturally occurring compounds enhance the antimicrobial activity of the nanoemulgels and augment their capacity to cure acute infections by permitting a deeper penetration into the epidermal layers. Comparing the antimicrobial properties of turmeric and neem nanoemulgels to traditional antimicrobial medications is a crucial step in understanding their therapeutic potential and synergistic effects against infections caused by microbes [26]. Development and assessment of topical nanoemulgels based on a combination of neem and turmeric extract is one possible substitute for antimicrobial therapy since the synergistic effect of neem and turmeric nanoemulgel has not been studied previously. This is in reaction to the growing concern about the emergence of antibiotic resistance and the pressing need to find efficient medicines to fight microbial infections. The combination of neem and turmeric extract used to make the nanoemulgel is also reasonably priced and widely accessible. The present study aims to utilize the synergistic antimicrobial properties of neem and turmeric extracts in a nanogel delivery system for topical application, improving local retention, stability, and therapeutic efficacy.

## 2. Results and Discussion

### 2.1. Antioxidant Activity of Turmeric and Neem Leaf Extract

**Figure 1:**
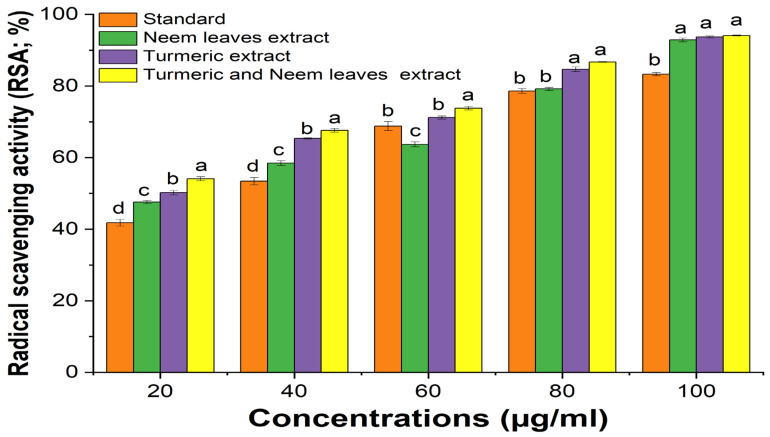
Antioxidant activity of the extracts. Different superscripted letters show the statistical significance values (*p*-value < 0.05) at the same concentration (µg/L). Also, ascorbic acid was used as standard.

The ability of DPPH to scavenge the free radical was observed by assessing the color change from purple to yellow measuring at a wavelength of 517 nm. The result was reported as radical scavenging activity (RSA). Significant scavenging capabilities were observed in the combination of turmeric and neem extract compared to the separate extract of turmeric and neem at different concentrations taking ascorbic acid as standard. Due to its synergistic effect, the turmeric–neem extract mixture’s antioxidant properties demonstrated noteworthy results, suggesting that the components of the extract are potent and effective antioxidants, and when combined, it enhances the activity (Figure 1, Table 1) [27,28].

### 2.2. Anti-Inflammatory Activity of Turmeric and Neem Leaf Extract

**Figure 2:**
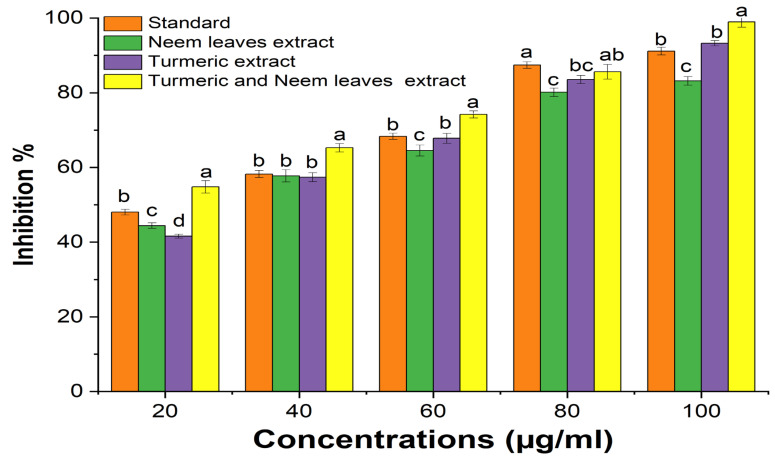
Anti-inflammatory activity of the extracts. Antioxidant activity of the extracts. Different superscripted letters show the statistical significance values (*p*-value < 0.05) at the same concentration (µg/L). Also, diclofenac sodium acid was used as standard.

The anti-inflammatory activity of the turmeric and neem leaf extract was evaluated and compared with the standard Diclofenac sodium. It was observed that both extracts showed notable anti-inflammatory properties when compared to the standard (Figure 2). The current investigation also shows that a mixture of turmeric and neem plant extract shows a synergistic effect where the anti-inflammatory property of the mixture was more significant than the individual extract’s ability to reduce inflammation depending on the concentration. Also, the plant extract may prevent hemolysis and more effectively stabilize the erythrocyte membrane at higher concentrations [27,29].

### 2.3. Antimicrobial Properties of Turmeric and Neem Leaf Extract

**Figure 3:**
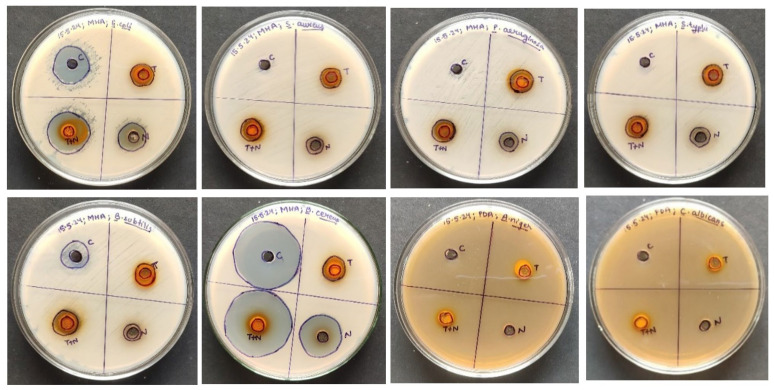
Antimicrobial activity of the extracts.

Using the agar well diffusion method, the antimicrobial capacity of the hydroalcoholic extract of neem leaves and turmeric was tested against a range of microorganisms, with Amoxycillin serving as a reference for bacterial pathogens and amphotericin B for fungal pathogens. The zone of inhibition indicates that there is a synergism between the turmeric and neem leaf extract mixture since it demonstrated considerably more antimicrobial activity than either extract alone (Table 2, Figure 3). Therefore, the extract’s antimicrobial action can be linked to the presence of phytochemicals with anti-pathogenic effects, including flavonoids, alkaloids, tannins, saponins, steroids, and phenolic compounds [30].

### 2.4. Turmeric- and Neem-Based Nanoemulsion Physicochemical Evaluation

#### 2.4.1. Color, Homogeneity, Phase Separation, Appearance, and pH

Physical parameters such as color, homogeneity, phase separation, and appearance were examined by visual inspection. No particle matter was found in either of the formulations because they were all clear and transparent (Table 3) [31]. Each product’s pH was measured using a digital pH instrument that was also validated; the results showed that the range of pH values was between 5 and 6, which is close to the pH range of the skin (Table 3) [32].

#### 2.4.2. Particle Characterization

**Figure 4:**
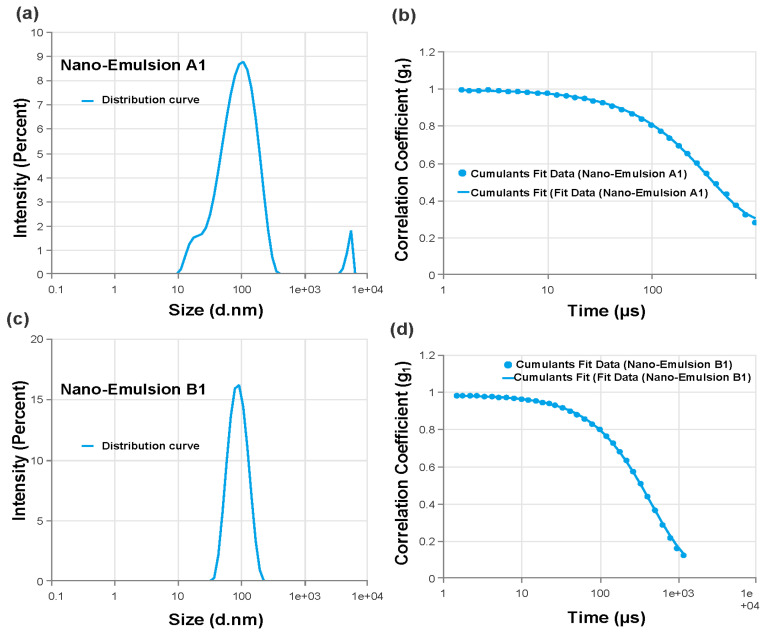
Size distribution by intensity of nanoemulsion under study. (**a**) Nanoemulsion A1’s, which contains 2% surfactant, distribution curve. (**b**) Correlation coefficient of nanoemulsion A1 shows cumulant data fit. (**c**) Nanoemulsion B1’s, which contains 4% surfactant, distribution curve. (**d**) Correlation coefficient of nanoemulsion B1 that shows cumulant data fit.

Each batch of nanoemulsion had droplet sizes ranging from 70 nm to 1768 nm. Formulations A1 (78.87 nm) and B1 (80.4 nm) had the smallest droplets. Every formulation under investigation had PDI values less than 0.691 (Table 4). The PDI values were also noticeably low (0.414 and 0.156, respectively) for the formulations with the smallest droplet sizes (A1 and B1), showing excellent stability and monodispersity as illustrated through the size distribution curves (Figure 4a,c). In addition, the B1 polydispersity index (PDI; a numerical value that represents the degree of variation in molecular mass or particle size within a polymer [33]) showed the lowest value (0.1569 ± 0.17). A PDI of less than 0.25 indicates a narrow size distribution like in the case of B1 compared to B5 [34,35]. The nanoemulsion A1 and B1 correlation coefficient distributions showed notable differences between A1 and B1 cumulant data fit curves (Figure 4b,d). Therefore, A1 and B1 were selected as the two representative nanoemulsion formulations for further research based on particle size and PDI values [32,33,34,35].

#### 2.4.3. Instrumental Analysis

**Figure 5:**
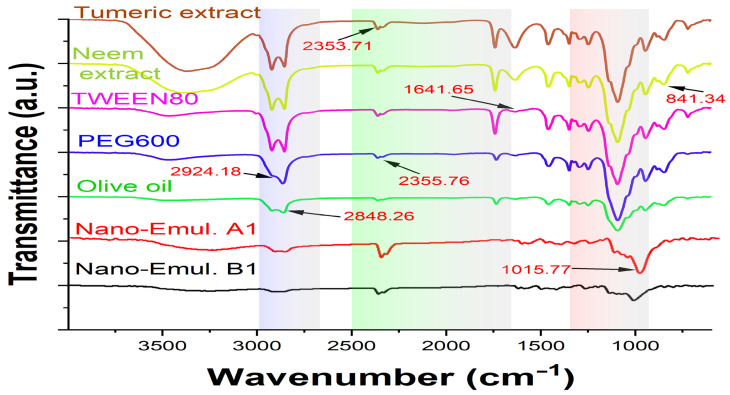
FTIR spectra of turmeric, neem extract, Tween 80, PEG600, olive oil, nanoemulsion A1, and nanoemulsion B1. The highlighted areas represent the different focused wavenumber ranges that explained the significant differences among the studied groups.

An FTIR analysis investigated the compatibility of turmeric and neem extract with olive oil, Tween 80, and PEG600 (Figure 5). The stretching of the benzene ring at 1600 cm^−1^, the O-H bonds at 3510 cm^−1^, the combination of C=O and C equivalent to C oscillations at 1600 cm^−1^, the C–H groups bending at 1430 cm^−1^, the C–O stretching at 1280 cm^−1^, and the C- oxygen-carbon stretching at 1025 cm^−1^ were the top values for both neem and turmeric [36]. The presence of water molecules causes an extra-wide band at around 3400 cm^−1^. Using the PEG 600, 1105 cm^−1^ (C–O–C bond), 3389–3420 cm^−1^ (OH stretch), and C–H stretching bands (2870–2890 cm^−1^) were detected, and for Tween 80 and olive oil, 2920 cm^−1^ (CH_3_ band), 2864 cm^−1^ (CH_2_-stretch), and 1095 cm^−1^ (C–O–C band) were detected. However, the O-H bond extension at 3447.10 cm^−1^ was the greatest of the formulation spectra, compared to 2870–2890 cm^−1^ (C–H stretch), 2864 cm^−1^ (CH_2_-stretch), and 1025 cm^−1^ (C–O–C stretching vibrations).

The retention of the polymer’s and other excipients’ characteristic peaks in the nanoemulsion formulations is confirmed by FTIR spectrum data, suggesting that the polymer and extracts’ constituent parts interact with one another on a molecular level. Moreover, the peaks exhibit no variation in the functional groups, indicating outstanding stability and the absence of phase separation in the formulation free from issues with extract–polymer compatibility [37].

### 2.5. Characterization of Turmeric- and Neem-Based Nanoemulgel

#### Physicochemical Properties

**Figure 6:**
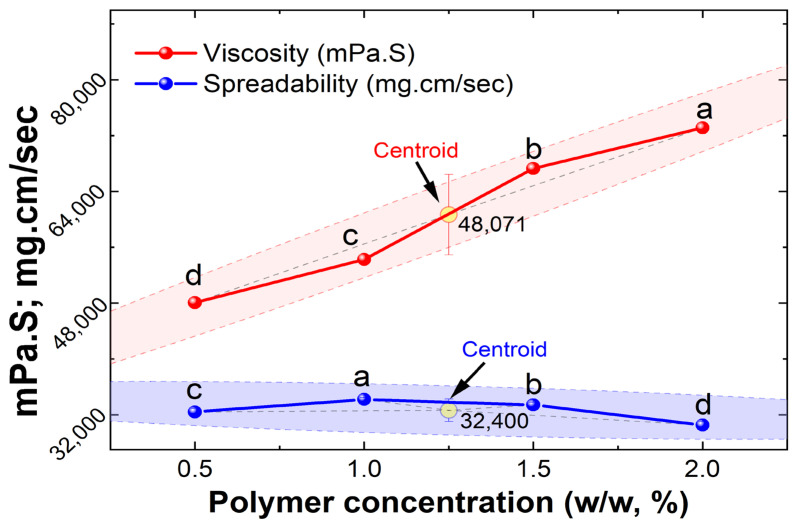
Impact of polymer content on nanoemulgel viscosity and spreadability. Different superscripted letters show statistical significance values (*p*-value < 0.05) in same line/analysis. The highlighted dashed areas represent the overall mean confidence levels, linear trendline, and centroid points of the examinal dataset for each analysis.

Physical characteristics including consistency, color, and homogeneity were examined by physical examination (Table 5) [38]. Based on viscosity and spreadability, optimization was performed on the nanoemulgel formulations, and it was found that when the polymer content increased, the nanoemulgels’ spreadability reduced (Table 5). In contrast to formulations F3 and F4, which had viscosities of 673,094 ± 3 and 73,127 ± 8 mPa·S, respectively, with low spreadability, F1 showed a viscosity of 48,072 ± 5 mPa·S with good spreadability 32 ± 0.5. Because of their ideal spreadability of 34 ± 0.2 and viscosity of 54,284 ± 7 mPa·S (Figure 6), formulations F2 and F1 were deemed excellent. Carbopol 934 is a synthetic polymer with a high viscosity of 30,000–50,000 centipoises and is commonly used in topical or transdermal formulations [39]. The viscosity of the nanoemulgel increased with the increase in the concentration of the gelling ingredient (Carbopol 934), and as a result, the formulation’s spreadability decreased. This is because the two properties of the formulation are inversely correlated [40].

Based on the nanoemulgel formulation optimization, it was observed that the F2 formulation was best optimized. The F2 formulation of the nanoemulgel was then incorporated with the best formulation of nanoemulsion (A1 and B1) prepared from herbal extracts for further studies (Table 6).

### 2.6. Drug–Excipient Interaction Studies

**Figure 7:**
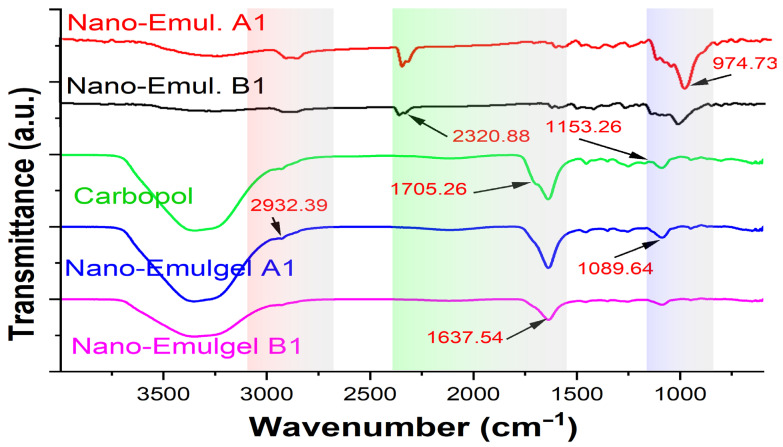
FTIR spectra of nanoemulsion A1, nanoemulsion B1, Carbopol 934, nanoemulgel A1, and nanoemulgel B1. The highlighted areas represent the different focused wavenumber ranges that explained the significant differences among the studied groups.

The drug–excipient compatibility in nanoemulgel formulations was determined using FTIR analysis (Figure 7). The C=O stretching vibration peaks of nanoemulgels A1 and B1 shifted to 1552 and 1545 cm^−1^, respectively, showing that hydrogen bonds are forming. Furthermore, the formulation’s wide OH peak moved to 3367 and 3344 cm^−1^, corresponding to the OH groups and hydrogen bonding between molecules. The C-H stretching was correlated with peaks that were found between 2853 and 2924 cm^−1^. Additionally, the formulation’s observation of the C-O-C stretching peak at 1049 cm^−1^ was changed to 1042–1043 cm^−1^. In Carbopol 934′s FTIR spectrum, the peak at 2927 cm^−1^ is associated with the compound CH_2_ stretching, the COOH group is connected to a peak at 1767 cm^−1^, and the COOH group is linked to peaks at 1451 cm^−1^ and 1246 cm^−1^ [41].

It is verified by FTIR spectrum data that the distinctive peaks of the polymer and other excipients are retained in the nanoemulgel formulations, indicating that there is intermolecular interaction between the components of the extracts and polymer. Furthermore, the peaks show no change in the functional groups depicting excellent stability and the absence of phase separation of the formulation devoid of extract–polymer compatibility issues [40].

### 2.7. Drug Permeation In Vitro Study Explaination

**Figure 8:**
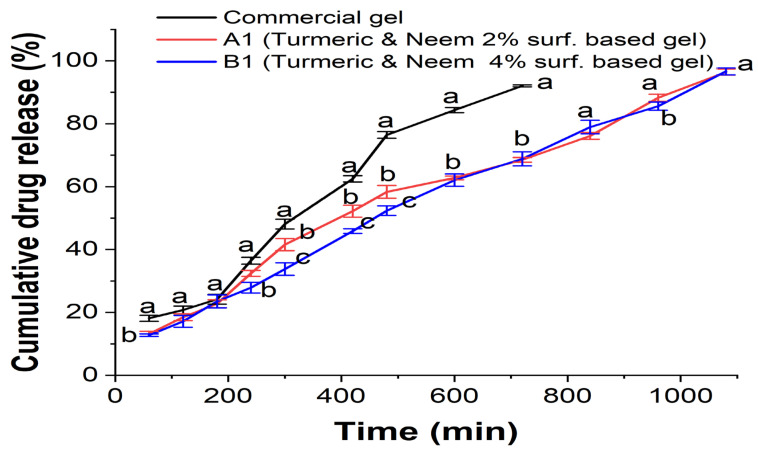
In vitro release study of nanoemulgel. Different superscripted letters show statistical significance values (*p*-value < 0.05) in same time interval (min) among commercial gel, A1, and B1.

In the drug permeation study, a dialysis membrane was employed in conjunction with the commercial hydrogel formulation to examine the release of the herbal extract from the nanoemulgel formulations (A1 and B1) (Figure 8). This is particularly noteworthy as the commercial gel is released less, and the extract was released for eighteen hours. With an increase in the content of Carbopol 934 in the nanoemulgels and nanoemulsions A1 and B1, the aggregate drug release increases from 96.4% (A1) to 96.6% (B1). The hydrophilic gel layer typically allows nanoemulgel to be released, and the rate of release is determined by polymer swelling, degradation, and diffusion.

Factors influencing drug release from carriers include the composition, ratio, and physical and chemical interactions between the extracts and the carriers [41,42]. With a reduced release rate, enhanced skin retention can be a more effective treatment for localized skin infections. The developed formulation path was complex and prolonged, which may have contributed to the decrease in the release rate of the nanoemulgels.

The released media that was used consisted of ethanol and distilled water (1:1 *v*/*v*) at pH 6.8. Oil droplets in nanoemulgel enter the skin’s subcutaneous layer directly after being released into the gel matrix, bypassing the hydrophilic phase change that occurs in nanoemulsion. Consequently, in subsequent formulations, formulation release patterns were reduced [43,44,45].

### 2.8. Ex Vivo Mucoadhesive Topical Administration Findings

Among the most important elements in the route of administration for topical delivery is mucoadhesion, which encourages prolonged release. Low adherence of Carbopol nanoemulgel was found. However, with the concentration of commercially available hydrogel, prepared nanoemulgel demonstrated superior mucoadhesive qualities (Table 7). Furthermore, when soaked in water, Carbopol swells a thousand times larger and has the strongest mucoadhesive property. This behavior might be explained by a longer polymeric chain and a more robust cohesive force between the goat skin and the specified nanoemulgel. Rheological considerations and mucoadhesive strength have a linear connection when compared to the product’s viscosity and mucoadhesive strength values. Within the bounds of our trials, it can be interpreted that there was a correlation between the formulation’s increased viscosity and its increased mucoadhesive strength [46]. Furthermore, it can be assumed that the swelling property of Carbopol might have helped in absorbing the exudates from the affected area.

### 2.9. Antimicrobial Properties of Nanoemulgel

**Figure 9:**
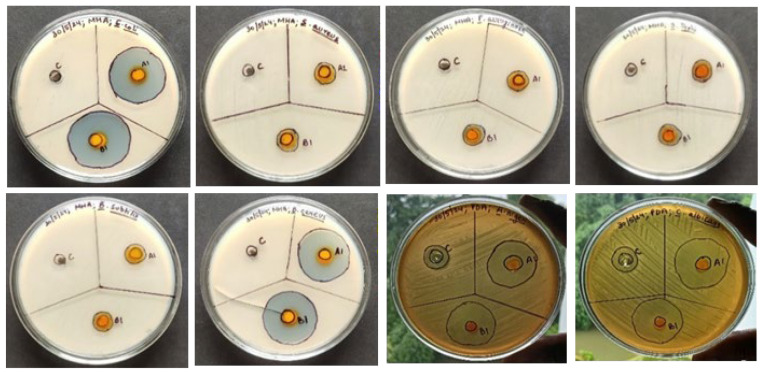
Antimicrobial activity of turmeric- and neem-leaf-based nanoemulgel.

The zone of inhibition indicates that the mixture of turmeric- and neem-leaf-based nanoemulgel substantial antibacterial activity was shown by the plant materials compared to the commercially marketed hydrogel against different microorganisms, taking the marketed hydrogel formulation (Megaheal) as a reference (Figure 9, Table 8) [47].

### 2.10. Stability Study

Characterization was performed on the optimized nanoemulgel formulation to ascertain its color, pH, homogeneity, viscosity, and spreadability. The optimized nanoemulgel formulation did not exhibit any notable alterations in its organoleptic qualities, and for three months, it stayed uniform at different temperatures such as the temperature in the refrigerator (4 °C ± 1 °C), ambient temperature (22–25 °C ± 1 °C), and incubated temperature (37 °C± 2 °C), as shown in Table 9 [48].

## 3. Conclusions

Currently, there has been an increased interest in preparing effective medication using natural products. In particular, the active constituents of herbal plants are used as potential sources having pharmacological properties including antimicrobial, anti-inflammatory, and antioxidant. Numerous research has been published that describes the formulation of nanoemulgel for transdermal delivery, such as bigels loaded with ciprofloxacin- and chitosan-laden nanoemulgel containing 5-fluorouracil. The preparation and evaluation of the nanoemulgel loaded with neem and turmeric extract for the topical delivery of drugs against microbial infection marks the conclusion of this investigation. According to reports, because of their target selectivity, non-toxicity, biocompatibility, and biodegradability, nanoemulsions are a promising choice for antimicrobial nanosystems. The improved nanoemulsion in this work was successfully blended with the gel foundation to develop a topical nanoemulgel formulation. The properties of the nanoemulgel loaded with neem and turmeric extract were successfully assessed to determine whether it was suitable for topical application. Effectiveness was observed in both formulations A1 and B1, suggesting that the proportion utilized is suitable for producing a useful nanoemulgel. The formulation that was optimized demonstrated a small droplet size and PDI within an ideal range. Following a three-month evaluation of the preparation for various stability tests, it was discovered that the formulations were stable, had excellent homogeneity, and showed no phase separation. Viscosity, spreadability, and drug content analysis were all found to be within an acceptable range. The formulations were next assessed for antimicrobial properties, and it was discovered that they had a significant zone of inhibition and were effective against a variety of pathogens. This zone of inhibition demonstrates how the neem- and turmeric-loaded nanoemulgel outperforms the separate extracts in terms of effectiveness and synergy. Furthermore, because of its mucoadhesive characteristics and prolonged release profile, it was determined that converting nanoemulsion into nanoemulgel would be more beneficial because of the latter’s prolonged release and increased duration of contact. Thus, the present study indicated that turmeric- and neem-extract-loaded nanoemulgel can be a promising alternative for microbial infection particularly associated with microorganisms via topical application, but for verification later on, an in vivo skin absorption study and cell-based assay might be helpful.

## 4. Materials and Methods

### 4.1. Chemicals, Plants, and Microbial Strains

All the chemicals, including Tween 80 (surfactant), PEG 600 (co-surfactant), olive oil, Carbopol 934 (polymer), ethanol, methanol, 2,2-Diphenul-1-Picrylhydrazyl (DPPH), triethanolamine, Amoxicillin, and Amphotericin-B, were of analytical grade. Megaheal gel (Aristo Pharmaceuticals Pvt. Ltd., Mumbai, India) was purchased from the market. Mueller Hinton Agar and Potato Dextrose Agar were obtained from HiMedia, Mumbai, India. The herbal plant sample was obtained from the Brainware University herbal garden which has been maintained by plants procured from the West Bengal State Medicinal Plants Board, Kalyani, India. The microbial strains, *Bacillus subtilis* (ATCC 6633), *Staphylocccus aureus* (ATCC 6538), *Escherichia coli* (ATCC 8739), *Pseudomonas aeruginosa* (ATCC 9027), *Bacillus cereus* (ATCC11778), *Salmonella typhi* (NCTC 786), *Aspergillus niger* (ATCC 16404), and *Candida albicans* (ATCC 10231), were gathered from the Central Drugs Laboratory (CDL) located in Kolkata, West Bengal, India.

### 4.2. Methods

#### 4.2.1. Extraction from Neem Leaves

Before ground into a powder, the neem leaves were thoroughly cleansed in distilled water and allowed to dry in the shade for 10 days. The extraction was done using methanol and distilled water in a proportion of 80:20 (*v*/*v*). The powdered sample was extracted for one hour at 25 °C and 150 rpm in a 500 mL scale using the hydroalcoholic mixture. After filtering the mixture using 125 mm Whatman filter paper, the extracts were further dried in a CHILLER SLIM-CHIL-300 rotary evaporator set to low pressure and 35 °C. In the culmination, a pale green residue was obtained and stored in an airtight container in a cool, dark place [49,50].

#### 4.2.2. Extraction from Turmeric Rhizomes

In a glass container containing 50 gm of powdered turmeric that had been bleached, 30 mL of ethanol and 70 mL of distilled water were added to produce a hydroalcoholic extract of the turmeric. Then, for 7 days, it was left to stand at room temperature (24 ± 1 °C) with frequent stirring. In order to extract the powder from the liquid, the suspension was filtered and then dried using the rotary evaporator. The extracts were thereafter stored in a refrigerator until they were needed [51].

#### 4.2.3. Antioxidant Activity of Hydroalcoholic Extract of Turmeric and Neem Leaves

##### DPPH Free Radical Scavenging Activity

The extracts’ capacity to scavenge free radicals was assessed using the DPPH test. A 0.1 mM DPPH solution was prepared in methanol. A total of 1.6 mL of hydroalcoholic extract of turmeric, neem, and a mixture of turmeric and neem at various mixture of concentrations (20, 40, 60, 80, and 100 µL) was prepared, with 2.4 mL of the DPPH solution prepared. After carefully mixing the concoction, it was allowed to sit at room temperature for about half an hour in a dark place. The absorbance of the combination was measured at 517 nm taking the standard as ascorbic acid. The percentage of inhibition was calculated with the following formula [27].

% of Inhibition = A_0_ − A_1_/A_0_ × 100, (1)

where A_0_ and A_1_ are the absorbance of the control and extract, respectively.

#### 4.2.4. Anti-Inflammatory Activity of Hydroalcoholic Extract of Turmeric and Neem Leaves

##### Bovine Serum Albumin Denaturation Activity

Inhibition of protein denaturation was ascertained to determine the anti-inflammatory efficacy. In this study, the test sample comprised 1% of bovine serum albumin (BSA) and various quantities of hydroalcoholic extracts of neem, turmeric, and a mixture of turmeric and neem at various concentrations (20, 40, 60, 80, and 100 µL). To correct the pH, 1 N of HCl was added. The reaction mixture was incubated in a water bath at 37 °C for 20 min and following that heated at 57 °C for 20 min. The reaction mixture was allowed to cool, and then its absorbance was measured at 570 nm using a UV-Vis spectrometer (UV-1900i, Shimadzu, Kyoto, Japan) taking Diclofenac sodium (Sigma-Aldrich, Raleigh, NC, USA) as standard. Using the following formula, the percentage inhibition of protein denaturation was calculated as follows:

% of Inhibition = (A_c_ − A_s_/A_c_ × 100) (2)

where A_c_ is the absorbance of the control, and A_s_ is the absorbance of sample [27].

#### 4.2.5. Antimicrobial Activity of Hydroalcoholic Extract of Turmeric and Neem Leaves

The antimicrobial activity of the hydroalcoholic extract of turmeric, neem, and a mixture of turmeric and neem was tested using agar well diffusion method with Mueller Hinton agar for antibacterial activity and Potato Dextrose agar to measure the antifungal activity against microorganisms like *Bacillus subtilis, Staphylococcus aureus*, *Escherichia coli*, *Pseudomonas aeruginosa*, *Bacillus cereus*, *Salmonella typhi*, *Aspergillus niger*, and *Candida albicans* [52]. Using sterile cotton swabs, the microorganisms were jabbed over the prepared Mueller Hinton and Potato Dextrose agar plate. Then, 10 µL of the produced hydroalcoholic extracts of turmeric, neem, and a mixture of turmeric and neem was placed into each well. The antibacterial and antifungal activity was assessed by measuring the zone of inhibition in triplicates during a 24 h incubation period at 37 °C [53].

#### 4.2.6. Preparation and Optimization of Turmeric- and Neem-Based Nanoemulsion

**Figure 10:**
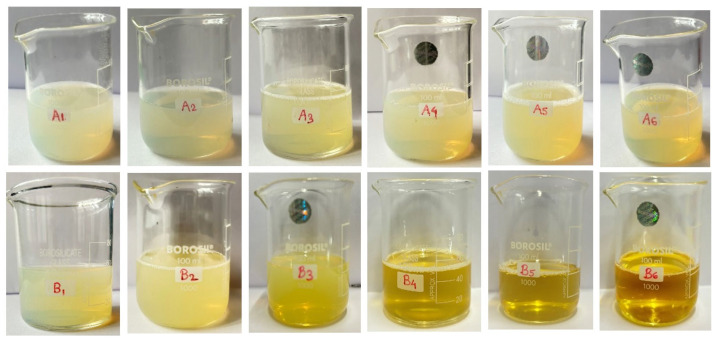
Formulation of nanoemulsion using turmeric and neem leaf.

Using a magnetic stirrer, the components of the oil phase of olive oil (1% *v*/*v*) and 1% *w*/*v* extracts of neem and turmeric were set for spontaneous formation of small oil droplets that occur at the boundary between the aqueous and organic phases under certain system conditions. The mixture was first mixed for 15 min. When the extracts in the oil phase had completely dissolved, 12 distinct combinations of oil (olive oil) and a mixture of surfactant (Tween 80) and co-surfactant (PEG 600) (Smix) were formed in different volume ratios to identify the stability differences (Table 10 and Table 11). Low-energy approaches utilized the intrinsic properties of the emulsifier, oil, and water systems to form nanoemulsions. Thus, both plant extracts in desired concentrations were combined with the oil phase, and in other hand-distilled water, they were combined with a 2:1 and 4:1 ratio of Smix [54,55]. With constant stirring, the aqueous phase was added drop by drop to the oil phase at 25 °C to produce the pre-emulsion (Figure 10). Then, high-shear homogenization (OV 625 Digital Disperser, Antylia Scientific, Monza, Italy) was used for 10 min at 10,000 rpm [56].

#### 4.2.7. Characterization of Turmeric- and Neem-Based Nanoemulsion

##### Physicochemical Properties and pH

Visual evaluations were conducted of the nanoemulsion’s color, homogeneity, consistency, and phase separation [57]. Using a digital validated pH meter at room temperature, the pH of the prepared nanoemulsion was ascertained [33].

##### Particle Characterization

The produced nanoemulsion formulation’s globule size was measured at 25 °C using Zetasizer ZTS1240 (Malvern Panalytical Ltd., Worcestershire, UK) [33,34].

##### FTIR Analysis

A Fourier transform infrared spectrophotometer (Nicolet 380 FT-IR Spectrometer, Madison, WI, USA) can be used for qualitative analysis and interaction investigations. This study aimed to evaluate the potential physiochemical interactions between the formulation ingredients used for preparing the nanoemulsion [58].

#### 4.2.8. Formulation of Turmeric- and Neem-Based Nanoemulgel

After being dissolved in water and allowed to swell overnight, Carbopol 934 was neutralized with triethanolamine. To transform the gel into emulgel, it was properly incorporated with the nanoemulsion using a magnetic stirrer and allowed to sit at room temperature (25 °C) for around 20 min [56]. Based on the gelling agent concentrations, spreadability, and viscosity, the formulation of nanoemulgel was optimized. Four distinct formulations with variable gelling agent concentrations (0.5–2%) were produced to optimize the formulation (Table 12). The final gel composition, which contained 1% (*w*/*w*) of turmeric–neem leaf extract, was created by mixing the components in the proper amounts.

#### 4.2.9. Characterization of Turmeric- and Neem-Based Nanoemulgel

##### Physical Examination

Color, homogeneity, and consistency of the resulting nanoemulgel were examined visually [59]. A digital validated pH instrument was used to confirm the pH of the nanoemulgel that was developed [33].

##### Rheological Studies

To determine the viscosity of the prepared nanoemulgel, a Brookfield viscometer (Brookfield Engineering Laboratories, Middleborough, MA, USA) was utilized. At 30 °C, the viscosity of the formulations was examined using spindles 1 and 2 rotated at 30 rpm for one minute, before the readings were recorded in triplicates [60].

#### 4.2.10. Spreadability of Nanoemulgel

The spreadability of the formulations was assessed using the Drag and Slip methodology [60] where two glass slides were used (7.5 cm long and 2.5 cm wide). The setup was composed of a wooden block with two glass slides (one slide is fixed, and the other one is movable) and a pulley fastened to a terminal. Subsequently, the formulation was mounted on the top fixed slide and pressed between the two plates to measure the spreadability of the mixture. To establish a homogenous layer of the formulation and release trapped air between the slides, the pulley’s upper glass slide has a 100 g weight fastened to it. The duration required for the upper/top slide to move was then recorded. The spreadability of the nanoemulgel was calculated using the formula below:

S = M × L/T (3)

where spreadability is S, weight or mass placed on the movable slide is M, glass slide length is L, and time taken to cover distance is T.

#### 4.2.11. FTIR Analysis

The FTIR analysis aimed to identify any incompatibilities between the active ingredients and excipients in the formulation of nanoemulgel, as previously mentioned [56]. An FTIR study was carried out for the nanoemulsion, nanoemulgel, and Carbopol 934.

#### 4.2.12. In Vitro Study

The release apparatus from Hanson Research was used for in vitro release. It had a working exterior area of 1.767 cm^2^ and a receptor capacity of 7.0 mL. As the releasing membrane, a dialysis cellulose membrane was used. The membrane was allowed to equilibrate with the receptor medium as an equal volume of ethanol and distilled water (1:1 *v*/*v*) at pH 6.8 for one hour before 1 g of nanoemulgel was gently added to it. In addition, Megaheal, a commercially available hydrogel (Aristo Pharmaceuticals Pvt. Ltd., Mumbai, India), was used as the control gel following the method of Kanekar et al. [61]. The composition of the commercially marketed hydrogel includes silver colloid (reported to have antimicrobial properties), carbomer (polymer), propylene glycol (to maintain the moisture), and triethanolamine (pH adjuster). A constant 37 ± 0.5 °C and 350 rpm were maintained for the releasing medium. At predefined intervals, after extracting the 2 mL sample, the same volume of media was added in its place [56]. At an effective wavelength of maximum absorbance of 431 nm, UV-visible spectrophotometry (UV-1900i, Shimadzu) was employed to estimate the extracted amount in every component [62].

#### 4.2.13. Ex Vivo Mucoadhesive Study

To determine the strength needed to extract the product from goat skin, an ex vivo mucoadhesive experiment was carried out using the Texture analyzer (Brookfield QTS). A nearby abattoir provided the fresh goat skin that was gathered. Once the skin had been attached to the lower probe, the top probe received nanoemulgel around 250 μL. When the top probe dropped at a constant rate of 0.3 mm/s, an acceleration of 0.4 N was generated at skin contact. The probe moved up after two minutes, measuring the detachment force [63].

#### 4.2.14. Antimicrobial Activity of Turmeric- and Neem-Based Nanoemulgel

Using Mueller Hinton agar and Potato Dextrose agar, the antibacterial activity was examined using the agar well diffusion method of the neem- and turmeric-based nanoemulgel, against microorganisms like *Bacillus subtilis*, *Staphylococcus aureus*, *Escherichia coli*, *Pseudomonas aeruginosa, Bacillus cereus*, *Salmonella typhi*, *Aspergillus niger*, and *Candida albicans*. Using sterile cotton swabs, the microorganisms were jabbed over the Mueller Hinton and Potato Dextrose agar plate. Then, 10 µL of the produced turmeric- and neem-based nanoemulgel, taking a marketed hydrogel (Megaheal, from Aristo Pharmaceutical Pvt. Ltd.) as control, was placed into each well. During a 24 h incubation period at 37 °C, to assess the antibacterial activity, the diameter of the zone of inhibition was measured three times [53,64,65].

#### 4.2.15. Stability Study

The physical stability of the formulation under accelerated storage circumstances was ascertained by conducting a stability analysis for the in situ formulation following the International Conference on Harmonization recommendations. The finished formulation was exposed to high temperatures and humidity levels. It was then stored for one to three months at various temperatures, including 4 °C ± 1 °C in the refrigerator, 22–25 °C ± 1 °C in the room, and 37 ± 2 °C in the incubator. Organoleptic alterations, physical stability, viscosity, and spreadability of the formulation were assessed at the end of the 30-, 60-, and 90-day periods [66].

#### 4.2.16. Statistical Analysis

All the results in tables and figures were used in triplicates and reported as averages ± standard deviation (SD). One-way analysis of variance (ANOVA) was applied among all the measurements, and *p*-value ≤ 0.05 was considered statistically significant by using SPSS 16.0 (IBM, USA) and Origin 2021 (Massachusetts, USA) software. Duncan and least significant difference (LSD) tests were calculated to measure the significance among the tested groups and properties.

## Figures and Tables

**Table 1:** Antioxidant activity of the extract.

Concentration (µg/mL)	Standard	% RSA (Neem Leaf Extract)	% RSA (Turmeric Extract)	% RSA (Turmeric and Neem Leaf Extract)
20	41.8 ± 0.92	47.6 ± 0.47	50.2 ± 0.59	54.1 ± 0.57
40	53.4 ± 1.02	58.45 ± 0.7	65.4 ± 0.21	67.6 ± 0.53
60	68.8 ± 1.26	63.71 ± 0.69	71.2 ± 0.42	73.8 ± 0.49
80	78.6 ± 0.65	79.2 ± 0.45	84.7 ± 0.68	86.7 ± 0.11
100	83.3 ± 0.47	92.87 ± 0.54	93.7 ± 0.28	94.1 ± 0.11

Values are expressed as Mean ± SD.

**Table 2:** Antimicrobial activity of the extracts.

Sr. No.	Pathogens	Zone of Inhibition (cm)			
		Standard *	Neem Leaf Extract	Turmeric Extract	Turmeric and Neem Leaf Extract
1	*Escherichia coli*	1.9 ± 1.041 ^b^	1.6 ± 0.194 ^c^	1.3 ± 1.053 ^d^	2.4 ± 1.064 ^a^
2	*Staphylococcus aureus*	-	1.2 ± 0.364 ^c^	2.5 ± 0.684 ^a^	1.6 ± 0.931 ^b^
3	*Pseudomonas aeruginosa*	-	1.8 ± 0.743 ^b^	2.4 ± 0.674 ^a^	2.0 ± 1.041 ^b^
4	*Salmonella typhi*	-	1.2 ± 0.764 ^b^	1.2 ± 0.640 ^b^	1.4 ± 0.131 ^a^
5	*Bacillus subtilis*	1.5 ± 0.661 ^a^	1.2 ± 1.163 ^a^	1.0 ± 0.319 ^b^	1.4 ± 0.452 ^a^
6	*Bacillus cereus*	3.4 ± 0.423 ^b^	1.3 ± 0.390 ^d^	2.4 ± 0.043 ^c^	3.6 ± 0.672 ^a^
7	*Aspergillus niger*	1.0 ± 0.981 ^a^	0.7 ± 0.714 ^bc^	0.5 ± 0.762 ^d^	0.8 ± 0.925 ^bc^
8	*Candida albicans*	1.1 ± 0.584 ^a^	0.7 ± 0.261 ^bc^	0.6 ± 0.431 ^d^	0.9 ± 0.335 ^bc^

Values are expressed as Mean ± SD. Different superscripted letters show the statistical significance values (*p*-value < 0.05) in the same row. * Amoxycillin was used as a standard antimicrobial agent.

**Table 3:** Physical evaluations of turmeric- and neem-leaf-based nanoemulsion.

Formulation Batch	Color	Phase Separation	Appearance	Homogeneity	pH Range
A1	Light Yellowish	Nil	Pellucid	Homogeneous	5.4 ± 0.13
A2	Light Yellowish	Nil	Pellucid	Homogeneous	5.0 ± 0.91
A3	Light Yellowish	Nil	Pellucid	Homogeneous	5.7 ± 0.62
A4	Light Yellowish	Nil	Pellucid	Homogeneous	5.7 ± 0.73
A5	Light Yellowish	Nil	Pellucid	Homogeneous	6.1 ± 0.44
A6	Light Yellowish	Nil	Pellucid	Homogeneous	6.3 ± 0.19
B1	Light Yellowish	Nil	Pellucid	Homogeneous	5.0 ± 0.53
B2	Light Yellowish	Nil	Pellucid	Homogeneous	5.0 ± 0.69
B3	Yellowish	Nil	Pellucid	Homogeneous	5.2 ± 0.33
B4	Yellowish	Nil	Pellucid	Homogeneous	6.2 ± 0.78
B5	Yellowish	Nil	Pellucid	Homogeneous	6.6 ± 0.57
B6	Yellowish	Nil	Pellucid	Homogeneous	6.8 ± 0.84

Values are expressed as Mean ± SD.

**Table 4:** Droplet sizes and PDI values of nanoemulsions.

Formulation Batch	Average Droplet Size (nm)	PDI
A1	78.87 ± 2.67 ^j^	0.4148 ± 0.130 ^b^
A2	102.3 ± 1.83 ^i^	0.4355 ± 0.098 ^b^
A3	119.6 ± 1.15 ^h^	0.3261 ± 0.055 ^b^
A4	139.3 ± 0.81 ^g^	0.256 ± 0.032 ^b^
A5	242.3 ± 2.56 ^f^	0.4787 ± 0.023 ^b^
A6	278.1 ± 2.31 ^e^	0.2664 ± 0.221 ^bc^
B1	80.4 ± 2.35 ^j^	0.1569 ± 0.107 ^c^
B2	244.8 ± 0.89 ^f^	0.65772 ± 0.238 ^ab^
B3	308.9 ± 2.53 ^d^	0.2542 ± 0.201 ^bc^
B4	472.1 ± 2.82 ^c^	0.7557 ± 0.112 ^a^
B5	811.5 ± 1.32 ^b^	0.661 ± 0.079 ^a^
B6	1768 ± 1.89 ^a^	0.3541 ± 0.041 ^b^

* Values are expressed as Mean ± SD. Different superscripted letters show the statistical significance values (*p*-value < 0.05) in the same column.

**Table 5:** Physical evaluations of nanoemulgel.

Formulation Batch	Color	Consistency	Homogeneity	pH	Viscosity (mPa·S)	Spreadability (g·cm/s)
F1	Yellowish	Good	Excellent	6.13 ± 0.01 ^a^	48,072 ± 5 ^a^	32 ± 0.5 ^a^
F2	Yellowish	Good	Excellent	6.3 ± 0.05 ^b^	54,284 ± 7 ^b^	34 ± 0.2 ^b^
F3	Yellowish	Good	Excellent	6.8 ± 0.05 ^d^	673,094 ± 3 ^c^	33 ± 0.3 ^c^
F4	Yellowish	Good	Excellent	6.6 ± 0.03 ^c^	73,127 ± 8 ^d^	30 ± 0.5 ^d^

* Values are expressed as Mean ± SD. Different superscripted letters show the statistical significance values (*p*-value < 0.05) in the same column.

**Table 6:** Formulation of extract-loaded nanoemulgel.

SI No.	Oil Phase (% *v*/*v*)	Turmeric Extract (%*w*/*v*)	Neem Leaf Extract (%*w*/*v*)	Surfactant (% *v*/*v*)	Co-surfactant (% *v*/*v*)	Carbopol 934 (% *w*/*w*)	Triethanolamine	Distilled Water (% *v*/*v*)
A1	1	1	1	2	1	1	q.s.	Upto 50 mL
B1	1	1	1	4	1	1	q.s.	Upto 50 mL

**Table 7:** Ex vivo mucoadhesive study of nanoemulgel.

Formulation	Viscosity (mPa·S)	Mucoadhesive Force (N)
Commercial gel	34,284 ± 7 ^c^	0.748 ± 0.033 ^c^
A1 (Turmeric- and neem-based gel)	51,317 ± 8 ^b^	2.235 ± 0.120 ^b^
B1 (Turmeric- and neem-based gel)	54,251 ± 4 ^a^	2.421 ± 0.128 ^a^

Values are expressed as Mean ± SD. Different superscripted letters show the statistical significance values (*p*-value < 0.05) in the same column.

**Table 8:** Antimicrobial activity of nanoemulgel.

Sr. No.	Pathogens	Zone of Inhibition (cm)		
		Commercial Gel	Turmeric- and Neem-Based Nanoemulgel (A1)	Turmeric and Neem Nanoemulgel (B1)
Antibacterial activity				
1	*Escherichia coli*	-	3.2 ± 0.672 ^b^	3.4 ± 0.762 ^a^
2	*Staphylocccus aureus*	-	3.4 ± 0.661 ^a^	3.3 ± 0.931 ^a^
3	*Pseudomonas aeroginosa*	-	1.3 ± 0.473 ^a^	1.3 ± 0.431 ^a^
4	*Salmonella typhi*	-	1.3 ± 0.264 ^a^	1.3 ± 0.440 ^a^
5	*Bacillus subtilis*	-	1.2 ± 0.163 ^a^	1.1 ± 0.319 ^b^
6	*Bacillus cereus*	-	3.4 ± 0.290 ^a^	3.2 ± 0.843 ^a^
Antifungal activity				
7	*Aspergillus niger*	1.4 ± 0.294 ^b^	2.4 ± 0.416 ^b^	2.7 ± 0.649 ^a^
8	*Candida albicans*	1.4 ± 0.682 ^b^	3.2 ± 0.068 ^a^	2.8 ± 0.301 ^b^

Values are expressed as Mean ± SD. Different alphabet superscripts indicate that values differ significantly (*p* < 0.05) in the same row.

**Table 9:** The optimized nanoemulgel formulation stability studies.

Formulation Batch	Color	Consistency	Homogeneity	pH	Viscosity (mPa·S)	Spreadability (g·cm/s)
A1	Yellowish	Good	Excellent	6.1 ± 0.4 ^a^	58,10 ± 2.00 ^b^	31.12 ±3.1 ^b^
B1	Yellowish	Good	Excellent	6.3 ± 0.2 ^a^	61,11 ± 0.04 ^a^	33.25 ± 2.2 ^a^

Values are expressed as Mean ± SD. Different superscripted letters show the statistical significance values (*p*-value < 0.05) in the same column.

**Table 10:** Preparation and optimization of nanoemulsion using turmeric and neem leaf extract with surfactant to co-surfactant 2:1 ratio.

SI No.	Oil Phase (% *v*/*v*)	Turmeric Extract (%*w*/*w*)	Neem Leaf Extract (%*w*/*w*)	Surfactant: Co-Surfactant 2:1		Distilled Water (% *v*/*v*)
				Surfactant (% *v*/*v*)	Co-Surfactant (% *v*/*v*)	
A1	1	1	1	2	1	Upto 50 mL
A2	1	1	1	3	1.5	Upto 50 mL
A3	1	1	1	4	2	Upto 50 mL
A4	1	1	1	5	2.5	Upto 50 mL
A5	1	1	1	6	3	Upto 50 mL
A6	1	1	1	7	3.5	Upto 50 mL

**Table 11:** Preparation and optimization of nanoemulsion using turmeric and neem leaf extract with surfactant to co-surfactant 4:1 ratio.

SI No.	Oil Phase (% *v*/*v*)	Turmeric Extract (%*w*/*w*)	Neem Leaf Extract (%*w*/*w*)	Surfactant: Co-surfactant (4:1)		Distilled Water (% *v*/*v*)
				Surfactant (% *v*/*v*)	Co-Surfactant (% *v*/*v*)	
B1	1	1	1	4	1	Upto 50 mL
B2	1	1	1	6	1.5	Upto 50 mL
B3	1	1	1	48	2	Upto 50 mL
B4	1	1	1	10	2.5	Upto 50 mL
B5	1	1	1	12	3	Upto 50 mL
B6	1	1	1	14	3.5	Upto 50 mL

**Table 12:** Composition of nanoemulgel.

Constituents	F1	F2	F3	F4
Carbopol 934 (% *w*/*w*)	0.5	1	1.5	2
Triethanolamine	0.1 mL	0.1 mL	0.1 mL	0.1 mL
Distilled water	q.s.	q.s.	q.s.	q.s.

## Data Availability

The original contributions presented in this study are included in the article.
